# IFN-related gene expression defines disease activity, organ involvement and treatment response in JDM

**DOI:** 10.1093/rheumatology/keag384

**Published:** 2026-07-25

**Authors:** Helena Codes-Méndez, Senne Cuyx, Aris E Syntakas, Afroditi Barmpakou, Elena Moraitis, Sandrine Compeyrot-Lacassagne, Muthana Al-Obaidi, Charalampia Papadopoulou

**Affiliations:** Rheumatology Department, Hospital de la Santa Creu i Sant Pau, Barcelona, Spain; Paediatric Rheumatology, Great Ormond Street Hospital for Children, NHS Foundation Trust, London, UK; Paediatric Rheumatology, Great Ormond Street Hospital for Children, NHS Foundation Trust, London, UK; Paediatric Rheumatology, Great Ormond Street Hospital for Children, NHS Foundation Trust, London, UK; Infection, Immunity and Inflammation Research and Teaching Department, UCL Great Ormond Street Institute of Child Health, London, UK; Paediatric Rheumatology, Great Ormond Street Hospital for Children, NHS Foundation Trust, London, UK; Paediatric Rheumatology, Great Ormond Street Hospital for Children, NHS Foundation Trust, London, UK; Infection, Immunity and Inflammation Research and Teaching Department, UCL Great Ormond Street Institute of Child Health, London, UK; Paediatric Rheumatology, Great Ormond Street Hospital for Children, NHS Foundation Trust, London, UK; Infection, Immunity and Inflammation Research and Teaching Department, UCL Great Ormond Street Institute of Child Health, London, UK; Paediatric Rheumatology, Great Ormond Street Hospital for Children, NHS Foundation Trust, London, UK; Infection, Immunity and Inflammation Research and Teaching Department, UCL Great Ormond Street Institute of Child Health, London, UK; Paediatric Rheumatology, Great Ormond Street Hospital for Children, NHS Foundation Trust, London, UK; Infection, Immunity and Inflammation Research and Teaching Department, UCL Great Ormond Street Institute of Child Health, London, UK

**Keywords:** JDM, IFN type-I, biomarkers, severity of illness index, disease activity, organ damage

## Abstract

**Objectives:**

To evaluate the relationship between IFN-related gene (IRG) expression and disease activity, organ involvement and serological subgroups in JDM and to assess its potential as a biomarker for disease monitoring and treatment response.

**Methods:**

Patients with JDM enrolled in a single-centre cohort were assessed using standardized clinical measures, including physician’s global assessment (PGA), Childhood Myositis Assessment Scale (CMAS), Manual Muscle Testing (MMT8) and modified skin disease activity score. Associations between IRG expression and clinical features were analysed using correlation analyses and mixed-effects models. Longitudinal trajectories of disease activity were explored using latent class mixed models.

**Results:**

Seventy-four patients contributed 158 samples. Expression of type-I IRGs (IFI27, IFI44L, IFIT1, RSAD2, SIGLEC1) and CXCL10 was significantly higher in active disease and correlated with muscle and skin disease activity. IRG expression inversely correlated with CMAS and MMT8 and positively with mDAS. Distinct organ-specific associations were identified: CXCL9 expression was associated with interstitial lung disease, while IL-18 was associated with calcinosis, cutaneous ulceration and gastrointestinal involvement. Higher IRG expression was observed in patients with anti-MDA5 and anti-NXP2 autoantibodies. Longitudinal analyses demonstrated that IRG expression paralleled disease activity trajectories and decreased in patients achieving inactive disease, including those treated with baricitinib.

**Conclusion:**

IFN pathway activation is closely associated with disease activity and organ involvement in JDM. IRG expression reflects dynamic changes in disease status and may serve as a clinically useful biomarker for monitoring disease activity, stratifying patients by organ involvement and supporting response assessment to targeted therapies.

*Rheumatology* key messages

- IFN-related gene expression closely reflects disease activity in JDM.
- Distinct IFN signatures correlate with specific organ involvement and myositis-specific autoantibodies.
- IFN-related gene expression dynamically decreases with disease improvement and targeted JAK inhibition.

## Introduction

JDM is a rare, chronic, immune-mediated myopathy of childhood characterized by inflammation of muscle, skin and small blood vessels [1–4]. Although advances in diagnosis and therapy have improved outcomes, the disease course remains heterogeneous and many patients continue to experience persistent or relapsing activity, resulting in cumulative tissue damage and functional impairment [5, 6]. Identifying reliable biomarkers that accurately reflect disease activity and organ involvement remains a key unmet need in paediatric rheumatology [4, 7–9].

Current disease assessment relies primarily on clinical measures, including the Childhood Myositis Assessment Scale (CMAS), Manual Muscle Testing (MMT8) and the modified skin disease activity score (mDAS), supplemented by laboratory markers such as muscle enzymes, CRP and ESR [2, 4, 10–12]. Although these methods are helpful, they may not detect mild inflammation and sometimes miss ongoing disease activity in patients on immunosuppressive treatment [7, 8]. For example, serum creatine phosphokinase (CPK) levels can be normal even when the disease is active [2, 3, 6, 13]. In view of these gaps, there is a need for molecular biomarkers that reflect immune activity and enhance current assessments [6, 7].

IFN signalling plays a key role in the development of JDM [1, 4, 14–17]. Type-I IFNs, like IFN-α and IFN-β, increase the activity of IFN-related genes (IRGs) that help fight viruses, control the immune system and cause inflammation [1, 18]. Studies have found that these genes are highly active in the blood and muscles of JDM patients, making a strong IFN signature a main feature of the disease [1, 4, 14–17]. Type-II IFN (IFN-γ) pathways also lead to immune problems and tissue damage, especially in cases with skin ulcers and lung disease [14–16, 19].

Even though these pathways are better understood now, they have not yet led to widely used clinical biomarkers [15, 20]. Higher IFN scores are linked to more severe disease and muscle weakness, but there is little information about how these scores relate to specific organs or how they change over time [4, 14–16]. Also, the different roles of type-I and type-II-IFN signalling in symptoms have not been fully studied [4, 14, 15]. Understanding these details could help doctors monitor and treat patients in a more personalized way [15, 20].

The development of Janus kinase (JAK) inhibitors, which block IFN signalling, highlights the need for strong IFN-based biomarkers [21–24]. Tracking IRG expression could help identify ongoing disease, measure how well treatments work and predict flares or remission [7, 15]. Using these biomarkers in regular care could make disease management more precise, especially for difficult cases [7, 20, 25].

In this study, we looked at how type-I and type-II IRGs are expressed in relation to disease activity in different organs in a large group of children with JDM. We also studied how these gene expressions relate to lab results and myositis-specific autoantibodies (MSAs), and we tracked changes in IRG expression during treatment. By combining molecular data with detailed clinical information, the potential of IRG expression as a JDM biomarker was assessed.

## Methods

### Study design and setting

This was a retrospective, cross-sectional study with longitudinal data available for a subset of patients, conducted at one of the largest paediatric rheumatology referral centres in Europe. Approval was obtained from the North-East Yorkshire Research Ethics Committee (MREC 01/3/022), and written informed consent was obtained from parents or legal guardians, with assent from participants where appropriate.

### Participants

Patients were eligible if they fulfilled the 2017 EULAR/ACR criteria or Peter and Bohan criteria for JDM [26–28]. Patient records from between May 2020 and June 2024 were reviewed for inclusion. Exclusion criteria included alternative connective tissue disease diagnoses, active infection at the time of sampling or insufficient RNA quality. During the study period, 10 patients were excluded after reclassification as having connective tissue diseases other than JDM, including SLE, SSc, mixed connective tissue disease or immune-mediated necrotizing myopathy. Demographic and clinical characteristics, including age at diagnosis, disease duration and organ involvement were recorded at each visit. Corticosteroid exposure at the time of each blood sampling was recorded and converted to prednisolone-equivalent doses, and concomitant immunosuppressive treatments were also documented.

### Clinical and laboratory assessment

Clinical assessment was performed by experienced paediatric rheumatologists. Global disease activity was scored using a physician’s global assessment (PGA) on a 10-cm visual analogue scale (VAS). Disease activity in specific organ systems was assessed using standardized outcome measures: CMAS and MMT8 for muscle activity and mDAS, including erythema, Gottron’s papules, heliotrope rash and ulceration for skin activity. Joint, pulmonary, gastrointestinal and constitutional involvement were recorded as either present or absent based on clinical evaluation and ancillary investigations (e.g. joint ultrasound, high-resolution CT thorax, swallowing studies). Disease was classified as either active or inactive based on PGA-VAS, EULAR/ACR core set measures [29, 30] and modified PRINTO criteria [31]. For longitudinal analyses, interval clinical status was categorized as stable, improving or worsening based on the treating physician’s global assessment, integrating changes in PGA-VAS, organ-specific disease activity and treatment escalation or de-escalation between consecutive visits.

Routine laboratory parameters were collected at each visit, including ESR, CRP, ferritin, CPK, lactate dehydrogenase (LDH), aspartate aminotransferase (AST), alanine aminotransferase (ALT) and complete blood count. Myositis-specific and myositis-associated autoantibodies (MSAs/MAAs) were detected using commercial immunoblot assays.

### Genetic sequencing and biomarker assessment

Peripheral blood was collected in PAXgene tubes, RNA was extracted, complimentary DNA (cDNA) was synthesized and quantitative real-time polymerase chain reaction (qPCR) was performed to quantify gene expression [32–34]. Expression of 10 IRGs was assessed as previously described [32] including the following transcripts: *CXCL9, CXCL10, IFI27, IFI44L, IFIT1, IFNB1, IFNG, RSAD2, SIGLEC1* and *IL18.*

### Statistical analysis

Continuous variables were summarized as median and interquartile range, and categorical variables as percentages. Normality was assessed using the Shapiro–Wilk test. Comparisons between groups were performed using Mann–Whitney *U* and Kruskal–Wallis tests, followed by pairwise *post hoc* analyses using Dunn’s test. Categorical variables were compared using Fisher’s exact test where appropriate. Bonferroni correction was applied to adjust for multiple testing. Correlations were assessed using Spearman’s correlation coefficients. Receiver operating characteristic (ROC) curves were generated to assess the discriminatory ability of individual genes for active vs inactive disease.

An IFN score was calculated for each sample as the geometric mean of the expression levels of all 10 IRGs. To further evaluate the association between disease activity parameters and the expression of the IRGs, while accounting for the longitudinal structure of the dataset, linear mixed-effects models were fitted using the *lme4* package [35]. Each model was adjusted for age and sex, and a random intercept for patient ID was included to account for within-patient correlation across repeated samples. Bonferroni correction was applied to mixed-model results to adjust for multiple comparisons.

Latent class mixed models were constructed using the *lcmm* package [36] to identify subgroups of patients with distinct trajectories of disease activity over time. Trajectory modelling was anchored to the first available IRG sampling timepoint for each patient rather than to the time of diagnosis. After determining the optimal number of classes for each model based on Akaike’s Information Criterion (AIC), IRG expression was compared across the derived trajectory classes to assess whether specific IRGs were differentially associated with particular longitudinal disease activity patterns.

Exploratory analyses were performed to assess differences in IRG expression and composite IFN scores according to baricitinib exposure status. For unpaired comparisons between samples collected during baricitinib treatment and those collected off treatment, the Mann–Whitney *U* test was used. Bonferroni correction was applied to adjust for multiple testing where appropriate.

To further evaluate longitudinal within-patient changes associated with baricitinib treatment, paired analyses were conducted in patients with available samples both before and during treatment. Given the variable number of samples per patient across time periods, the median expression value for each IRG and for the composite IFN score was calculated separately for the pre-treatment and on-treatment periods for each individual patient. Paired comparisons were then performed using the Wilcoxon signed-rank test.

A *P*-value <0.05 was considered statistically significant. All analyses were conducted using R version 4.3.2 (R Foundation for Statistical Computing, Vienna, Austria) and SPSS version 26 (IBM Corp., Armonk, NY, USA).

## Results

### Patient characteristics and disease status at sampling

A total of 74 patients with JDM were included, contributing 158 peripheral blood samples. Longitudinal data were available for 41 patients who provided more than one sample over the study period. Twenty-one patients (28%) had samples obtained at diagnosis, prior to or at initiation of immunosuppressive therapy. The clinical and immunological characteristics of the 74 patients are summarized in Table 1, reflecting the heterogeneous disease spectrum seen in a tertiary referral centre.

**Table 1:** Patient characteristics.

	Patients, *N* = 74
JDM diagnosis	
JDM	61 (82%)
JDM overlap	13 (18%)
Age at diagnosis, years, median (IQR)	7 (4–9)
Disease duration, years, median (IQR)	4.6 (3.5–8.1)
Sex, male:female	22:52
Time from disease onset to sample, days, median (IQR)	690 (20–2200)
Race and ethnicity	
White British	27 (36%)
White/European	7 (9%)
Black/African	6 (8%)
Black/Caribbean	2 (3%)
Black	3 (4%)
Black African/White	1 (1%)
Black Caribbean/White	2 (3%)
Asian	4 (5%)
Indian	1 (1%)
Asian/White	1 (1%)
Other	14 (19%)
Not stated	5 (7%)
ANA status	
Positive	57 (77%)
Negative	15 (20%)
Not done	2 (3%)
Myositis-specific autoantibodies (MSAs)	
MSA positive	41 (55%)
MSA negative	33 (45%)
MSA specificity^a^	
Anti-TIF1γ	14 (18.9%)
Anti-NXP2	10 (13.5%)
Anti-MDA5	9 (12.2%)
Anti-Mi2	6 (8.1%)
Anti-SRP	2 (2.7%)
Anti-SAE	1 (1.4%)
Anti-PL7	1 (1.4%)
Anti-HMGCR	1 (1.4%)
Myositis-associated autoantibodies (MAAs)	
MAA positive	20 (27%)
MAA negative	54 (73%)
MAA specificity^a^	
Anti-PmScl	10 (13.5%)
Anti-Ro52	7 (9.5%)
Anti-U1RNP	1 (1.4%)
Anti-Ku	1 (1.4%)
Clinical features at time of sample	
Gottron’s rash/papules	40 (54%)
Heliotrope rash	22 (30%)
Nailfold capillary changes	29 (40%)
Calcinosis	11 (15%)
Dysphonia	2 (3%)
Dysphagia	18 (24%)
Abdominal pain	10 (14%)
Interstitial lung disease	14 (19%)
Arthritis	17 (23%)
Treatment at time of sample (% of samples, *n* = 158)	
No treatment	22 (13.8%)
Any treatment	137 (86.2%)
Corticosteroids	
On corticosteroids	59 (37.1%)
Pulse corticosteroids (≥250 mg/day)	4 (2.5%)
Oral maintenance dose, mg/day, median (IQR)	15 (5–40)
Immunosuppressants	
MTX	69 (43.4%)
HCQ	74 (46.5%)
IVIG	62 (39.0%)
Mycophenolate mofetil	36 (22.6%)
Baricitinib	27 (17.0%)
Adalimumab	18 (11.3%)
Rituximab	10 (6.3%)
Cyclophosphamide	8 (5.0%)
Azathioprine	3 (1.9%)
Ciclosporin	2 (1.3%)
Disease activity at time of sample (% of samples, *n* = 158)	
Active disease	125 (79.0%)
Clinically inactive disease	33 (21.0%)

Values are *n* (%) unless otherwise specified.

a MSA and MAA specificity percentages are calculated over the total cohort (*N* = 74); two patients had dual MSA positivity. Treatment data are expressed as percentage of total samples (*n* = 158). Corticosteroid dose is expressed as prednisolone equivalent dose.

Abbreviations: ANA: antinuclear antibody; ILD: interstitial lung disease; IQR: interquartile range; IVIG: intravenous immunoglobulin.

The median age at sampling was 12 years (IQR 8–15), and 52 patients (70.3%) were female. Median age at diagnosis was 7 years (IQR 4–9), with a median disease duration of 4.6 years (IQR 3.5–8.1) at enrolment.

At the time of sampling, the majority of samples (79%) were obtained during clinically active disease, including 28% during active but stable disease, 30% during an improving disease course and 21% during worsening disease activity. Samples were obtained both close to diagnosis and during established disease, reflecting the referral-centre nature of the cohort.

All patients had a history of muscle and skin involvement. Interstitial lung disease (ILD) was present in 14 patients (19%), gastrointestinal involvement in 18 (24%) and calcinosis in 15% at the time of sampling. No cardiovascular involvement was observed.

MSAs were identified in 41 patients (55%). The most frequent specificities were anti-TIF1γ, anti-MDA5, anti-NXP2 and anti-Mi-2, while 44% of patients were seronegative.

At sampling, most patients were receiving immunosuppressive therapy, most commonly corticosteroids, HCQ and MTX. Fourteen patients (27 samples) were receiving baricitinib.

### IFN gene expression and global disease activity

**Figure 1:**
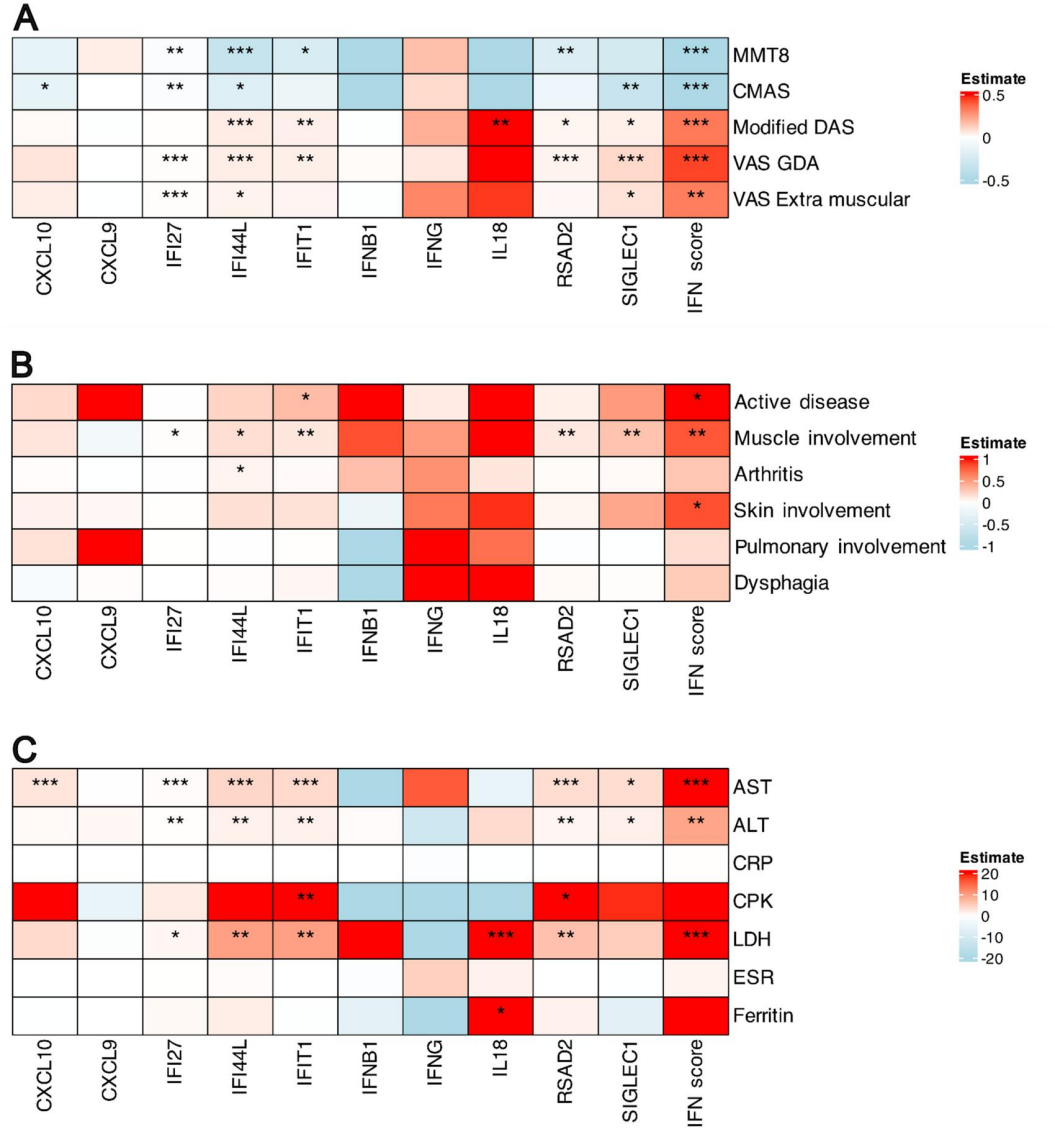
Effect of IFN-related gene expression on disease activity parameters. (A) Associations between IFN-related gene expression and continuous disease activity measures (MMT-8, CMAS, mDAS, physician global disease activity VAS, and extramuscular disease activity VAS). (B) Associations between IFN-related gene expression and categorical clinical features (active disease and muscle, gastrointestinal, joint, and skin involvement). (C) Associations between IFN-related gene expression and laboratory parameters (AST, ALT, CRP, CPK, LDH, ferritin, and ESR). Data presented show the effect of IRG expression on different disease activity parameters, estimated using a mixed-effects model (clinical variable = IFN gene + gender + age at sampling + [1 | patient ID]). IFN score was calculated using all IFN-related genes included in the panel, whereas the type-I IFN score was restricted to type-I IFN-inducible genes. MMT8: Manual Muscle Testing of eight muscular groups; CMAS: Childhood Myositis Assessment Scale, modified DAS: disease activity score for skin involvement; VAS: visual analogue score; GDA: global disease activity, AST: aspartate aminotransferase, ALT: alanine aminotransferase; CPK: creatine phosphokinase; LDH: lactate dehydrogenase.

Expression of type-I IFN-inducible genes (*IFI27*, *IFI44L*, *IFIT1*, *RSAD2* and *SIGLEC1*) and the IFN-inducible chemokine *CXCL10* were significantly higher in samples obtained during active disease compared with inactive disease (all *P* < 0.015). In contrast, *CXCL9*, *IFNB1*, *IFNG* and *IL18* expression did not differ significantly between active and inactive disease states (Table 2, Fig. 1).

**Table 2:** Association between the expression of each IFN gene and disease activity features.

	IFN genes expression									
	CXCL10	CXCL9	IFI27	IFI44L	IFIT1	IFNB1	IFNG	IL18	RSAD2	SIGLEC1
DA	0.013	N/S	<0.001	<0.001	<0.001	N/S	N/S	N/S	<0.001	<0.001
VAS-GDA (all)	N/S	N/S	N/S	N/S	N/S	0.022	N/S	N/S	N/S	N/S
VAS-GDA (active)	N/S	N/S	N/S	N/S	N/S	0.003	N/S	N/S	N/S	N/S
MUSCLE DA	0.007	N/S	<0.001	<0.001	<0.001	N/S	N/S	0.016	<0.001	<0.001
MMT8	0.004	N/S	<0.001	<0.001	<0.001	0.071	N/S	0.016	<0.001	<0.001
CMAS	0.030	N/S	<0.001	<0.001	<0.001	N/S	N/S	N/S	<0.001	<0.001
Arthritis	N/S	N/S	0.005	0.012	N/S	N/S	N/S	N/S	N/S	0.050
Skin DA	N/S	N/S	<0.001	<0.001	<0.001	N/S	N/S	N/S	<0.001	<0.001
Calcinosis	N/S	N/S	N/S	N/S	0.039	N/S	N/S	0.030	0.023	N/S
Ulceration	N/S	N/S	N/S	N/S	N/S	N/S	N/S	0.046	N/S	N/S
mDAS	0.020	N/S	<0.001	<0.001	<0.001	N/S	N/S	0.003	<0.001	<0.001
Lung DA	N/S	0.010	<0.001	0.011	0.034	N/S	N/S	N/S	0.016	0.028
Ever ILD	N/S	0.027	N/S	N/S	N/S	N/S	N/S	N/S	N/S	N/S
GI DA (dysphagia)	N/S	N/S	0.038	0.008	0.015	N/S	N/S	0.022	0.043	0.022

Abbreviations: CMAS: Childhood Myositis Assessment Scale; DA: disease activity (active/inactive); GI: gastrointestinal; ILD: interstitial lung disease; mDAS: modified disease activity score for skin involvement; MMT8: Manual Muscle Testing of eight muscular groups; N/S: non-significant; VAS-GDA: visual analogue score—global disease activity.

Several IRGs were associated with constitutional features of disease activity. The presence of systemic symptoms, including fever, weight loss, fatigue and functional impairment, was associated with increased expression of *IFI27*, *IFI44L*, *IFIT1*, *RSAD2*, *SIGLEC1* (all *P* < 0.001) and *CXCL10* (*P* = 0.015). Fatigue demonstrated similar associations except for *CXCL10*.

Correlation analyses using the physician’s global assessment (PGA-VAS) demonstrated that *IFNB1* was the only IRG significantly correlated with global disease activity, both across the entire cohort and when restricting analyses to clinically active patients.

### Musculoskeletal involvement

Patients with active muscle disease demonstrated significantly higher expression of *IFI27*, *IFI44L*, *IFIT1*, *RSAD2* and *SIGLEC1* (all *P* < 0.001), as well as *CXCL10* and *IL18* (Table 2). These genes showed strong negative correlations with both CMAS and MMT8 scores, indicating higher IFN pathway activation in association with active muscle inflammation and reduced muscle strength and function.

Respiratory muscle weakness was observed in 16 samples (10.1%) and was significantly associated with *IFI27* expression (*P* < 0.001), while no consistent associations were observed for other IRGs.

Arthritis was associated with increased expression of *IFI27*, *IFI44L* and *SIGLEC1*. These musculoskeletal associations were observed across samples obtained both at diagnosis and during established disease.

### Cutaneous involvement

Cutaneous disease activity assessed by the mDAS correlated positively with expression of multiple IRGs, including *IFI27*, *IFI44L*, *IFIT1*, *RSAD2*, *SIGLEC1*, *CXCL10* and *IL18* (Table 2). Among these, *IL18* showed the strongest association with vasculopathic and damage-related skin features.

Analysis of individual cutaneous manifestations demonstrated overlapping but distinct IFN signatures (Supplementary Table S1). Extensive erythema and erythroderma were primarily associated with *IL18* expression. Calcinosis and cutaneous ulceration were significantly associated with *IL18*, with additional associations observed for *IFIT1* and *RSAD2* in ulcerative disease. Periungual capillary loop abnormalities assessed by nailfold video capillaroscopy were associated with the expression of multiple IRGs including *IL18*.

Rare cutaneous manifestations, including mucosal involvement and subcutaneous oedema, were associated with *CXCL9* and *IFNB1* expression, while no significant associations were observed for livedo reticularis or linear/extensor erythema.

### Pulmonary and gastrointestinal involvement

ILD was significantly associated with increased expression of *CXCL9*, a type-II IRG (*P* = 0.010), as well as *IFI27*, *IFI44L*, *IFIT1*, *RSAD2* and *SIGLEC1* (Table 2).

Gastrointestinal involvement, including dysphagia, was associated with increased *IL18* expression (*P* = 0.022), along with modest increases in several type-I IRGs (*P* < 0.05).

### Associations with laboratory disease activity markers

IRG expression correlated positively with multiple laboratory markers of inflammation and tissue injury. *IFI27* expression was associated with anaemia (*P* = 0.003), while lymphopenia showed strong associations with *CXCL9* (*P* = 0.003), *IFNB1* (*P* = 0.027), *IFI27*, *IFI44L*, *IFIT1*, *IL18*, *RSAD2* and *SIGLEC1* (all *P* < 0.001). No significant associations were observed for leukopoenia, neutropenia or thrombocytopenia.

Inflammatory markers demonstrated consistent associations with IFN pathway activation. ESR correlated with *IFI27* (*P* = 0.001) and *IFI44L* (*P* = 0.013), CRP correlated with *CXCL10* (*P* = 0.011) and ferritin levels were strongly associated with multiple IRGs, including *IFI27* and *IFI44L* (both *P* < 0.001), *IFIT1* and *SIGLEC1* (both *P* = 0.002), *RSAD2* (*P* = 0.001) and *CXCL10* (*P* = 0.038).

Markers of muscle injury demonstrated robust correlations with several IRGs: CPK correlated with *CXCL10* (*P* = 0.011), *IFI27* (*P* = 0.036), *IFI44L* (*P* = 0.023), *IFIT1* (*P* = 0.043), *RSAD2* (*P* = 0.039) and *SIGLEC1* (*P* = 0.022), while LDH showed highly significant associations with *CXCL10*, *IFI27*, *IFI44L*, *IFIT1*, *RSAD2* and *SIGLEC1* (all *P* < 0.001). Similarly, liver transaminases (AST and ALT) demonstrated associations with *CXCL10* (*P* = 0.016), *IFI27* and *IFIT1* (both *P* = 0.001), *IFNG* (*P* = 0.017), *IFI44L*, *RSAD2* and *SIGLEC1* (all *P* < 0.001). These associations remained significant in mixed-effects models accounting for age, sex and repeated sampling for longitudinal variability (Fig. 1).

### IFN-related gene expression and myositis-specific autoantibodies

**Figure 2:**
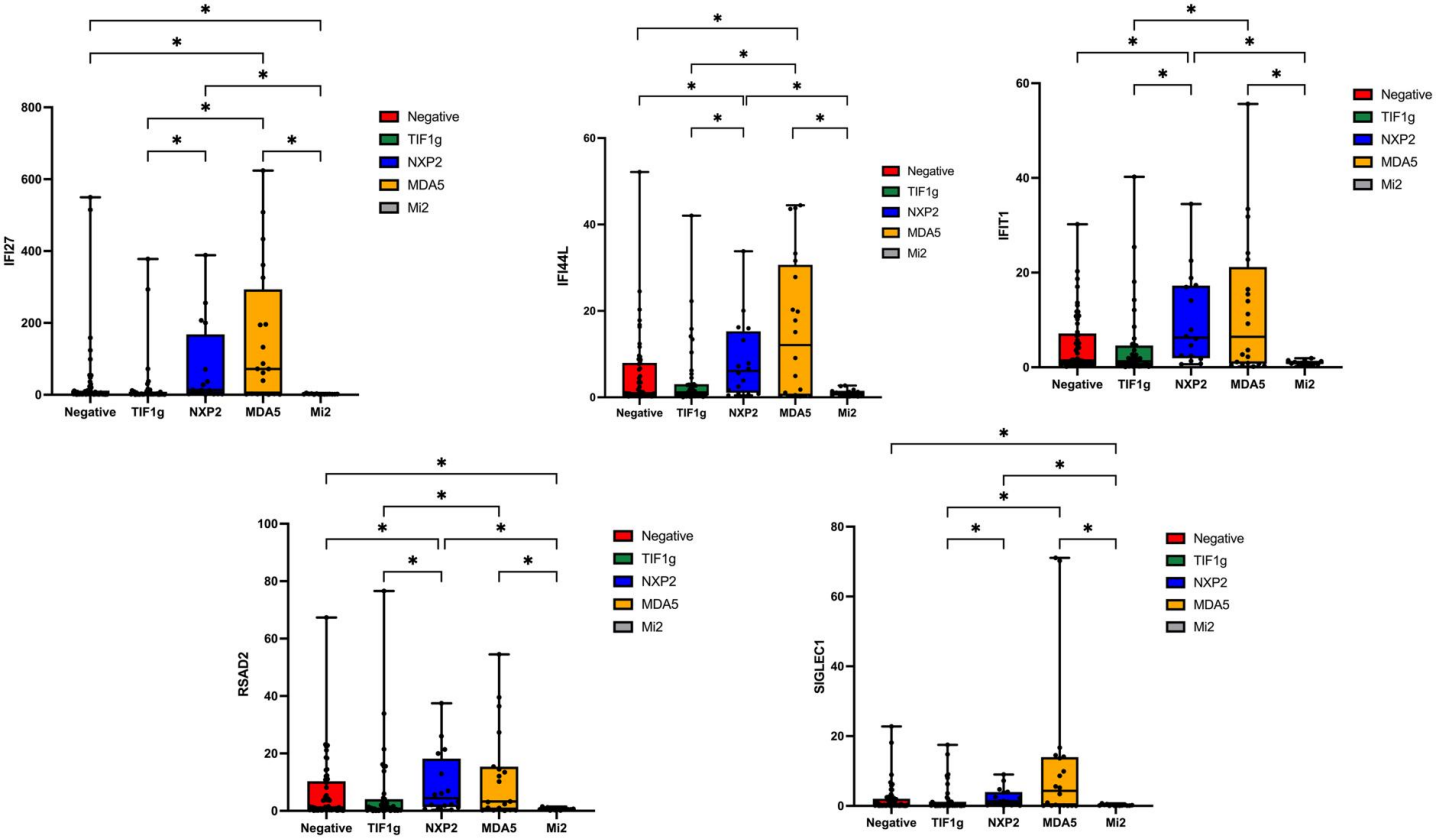
IFN-related gene expression according to myositis-specific autoantibody profile. Data are presented as box-and-whisker plots indicating median, interquartile range and outliers. Group comparisons were performed using the Kruskal–Wallis test with Bonferroni correction applied within the overall test framework to account for multiple comparisons. *P < 0.05, **P < 0.01.

Distinct IRG expression patterns were observed across MSA subgroups (Fig. 2). Patients with anti-MDA5 and anti-NXP2 antibodies demonstrated the highest overall IRG expression, particularly for *IFI27* (*P* = 0.001), *IFI44L* (*P* = 0.043), *IFIT1* (*P* = 0.004), *RSAD2* (*P* = 0.023) and *SIGLEC1* (*P* = 0.048). In contrast, patients with anti-TIF1γ and anti-Mi-2 antibodies exhibited lower and more heterogeneous IFN signatures, while seronegative patients showed the lowest overall IFN activity.

### Longitudinal analyses

**Figure 3:**
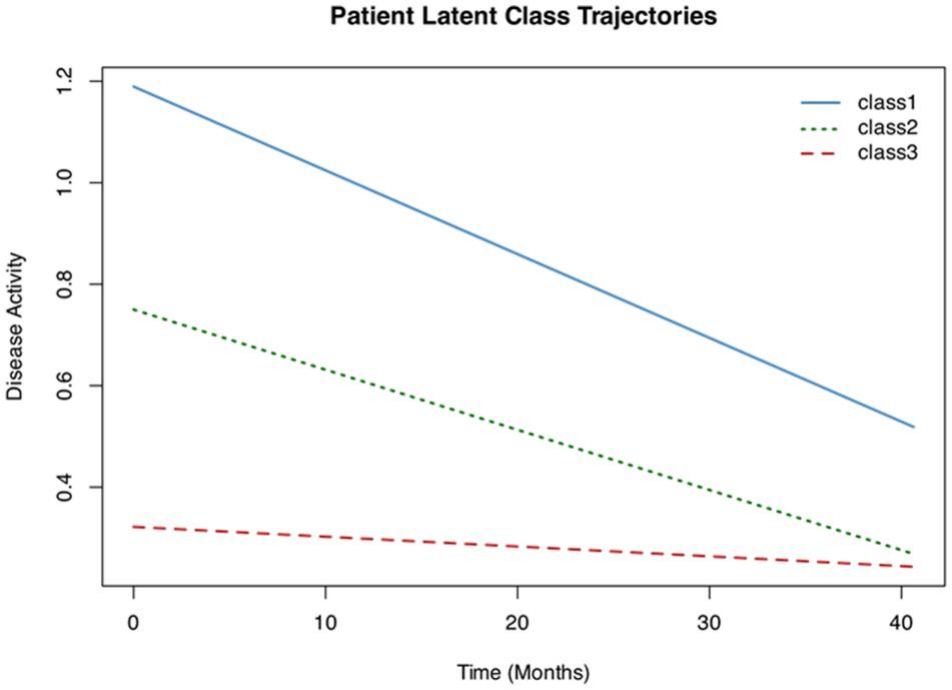
Longitudinal modelling of disease activity trajectories. Three distinct trajectories were identified through longitudinal modelling of disease activity, based on binary disease activity status (active vs inactive) and Physician Global Assessment—Visual Analogue Score (PGA-VAS). Class 1 included patients with active disease showing partial improvement over time. Class 2 comprised patients with low disease activity who progressed to inactive disease, Class 3 consisted of patients with sustained inactive disease

Forty-two patients contributed serial samples over time, including 21 patients with two samples, 10 with three samples and 11 with four or more samples; 21 patients had baseline samples obtained at diagnosis. Longitudinal modelling of disease activity trajectories, based on the binary disease activity status (active vs inactive) and the PGA-VAS as classifiers, identified three distinct patient classes (Fig. 3). The first class comprised patients with persistently active disease with partial clinical improvement over follow-up (*n* = 8 patients, 32 timepoints). The second class included patients with low disease activity at baseline who subsequently achieved inactive disease (*n* = 6 patients, 16 timepoints). The class group consisted of patients with persistently inactive disease over time (*n* = 28 patients, 79 timepoints).

IRG expression paralleled these disease activity trajectories (Supplementary Table S2). Patients in the persistently active group demonstrated the highest and most sustained expression of type-I IFN-inducible genes, including *IFI27*, *IFI44L*, *IFIT1*, *RSAD2* and *SIGLEC1*. In contrast, patients with persistently inactive disease showed consistently low IRG expression across all timepoints. Patients transitioning from active or low disease activity to inactive disease exhibited a marked decline in IRG expression over time, paralleling clinical improvement. These longitudinal changes were observed both in patients sampled close to diagnosis and in those with established disease. In treatment-naïve patients with paired diagnostic and follow-up samples available (*n* = 8), IRG expression decreased following treatment initiation, although this change did not reach statistical significance.

Among patients receiving baricitinib, a pronounced reduction in IRG expression was observed within 3–6 months of treatment initiation. No patients developed new organ involvement during follow-up, and no paradoxical increases in IRG expression were observed during clinical remission.

### Baricitinib treatment and IFN-related gene dynamics

An exploratory analysis was performed comparing IRG expression between samples from patients receiving baricitinib and samples from patients who were not receiving any systemic immunomodulatory treatment at the time of sampling. Fourteen patients contributed 27 samples collected during baricitinib exposure. No statistically significant differences in individual IRG expression or composite IFN scores were observed between the groups after correction for multiple testing (Supplementary Table S3). Based on unadjusted analyses, IFNB1 expression was lower in baricitinib-treated samples (*P* = 0.015).

**Figure 4:**
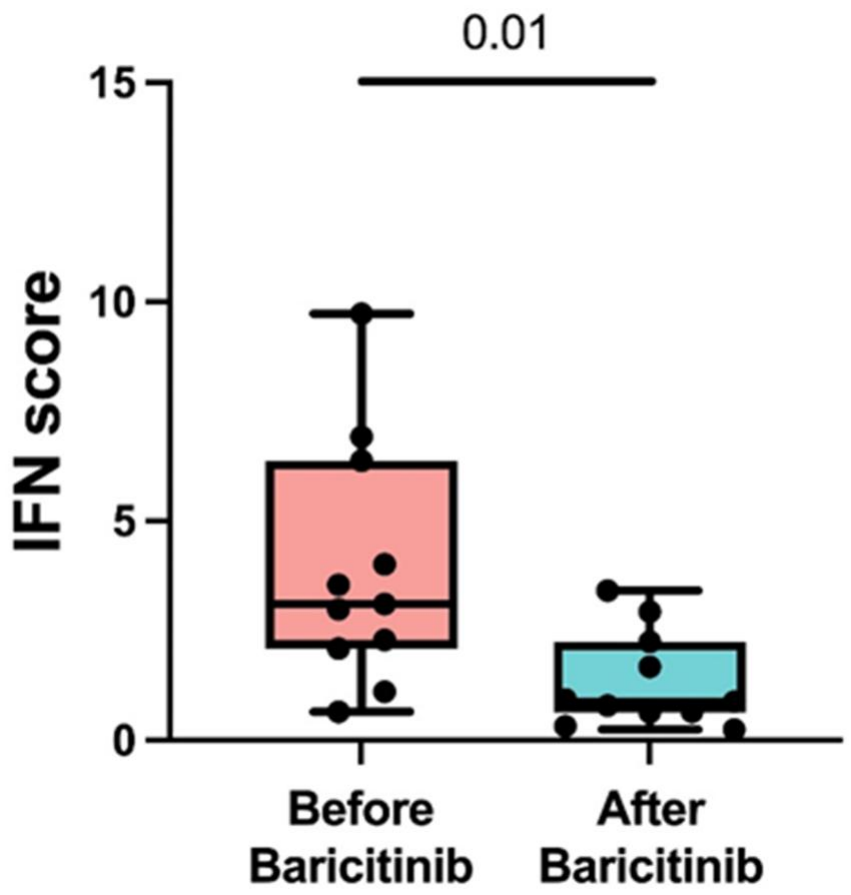
Changes in IFN-related gene expression after baricitinib initiation. Data are presented as box-and-whisker plots indicating median, interquartile range and outliers. Comparisons were performed using the Wilcoxon signed-rank test

To further assess longitudinal treatment-associated changes, within-patient analyses were performed in the subgroup of patients with paired pre-treatment and on-treatment samples. Among the 14 patients treated with baricitinib, 11 had samples available both before and during treatment. Most type-I IFN-related genes demonstrated reductions towards normalization following baricitinib initiation, including the composite IFN score (Fig. 4, Supplementary Table S4). Reductions were particularly notable for IFI27 (*P* = 0.010), IFI44L (*P* = 0.021), RSAD2 (*P* = 0.021) and SIGLEC1 (*P* = 0.010) expression. These molecular changes paralleled clinical improvement over time.

## Discussion

This study shows that activation of the IFN pathway, mainly type-I with some input from type-II-related mediators, is a reliable molecular marker of disease activity in JDM. In a large cohort of patients followed over time, expression of IRGs, especially *IFI27*, *IFI44L*, *IFIT1*, *RSAD2* and *SIGLEC1*, was closely associated with overall disease activity and multiorgan involvement. IRG expression decreased as patients improved and responded to treatment, suggesting its potential as a biomarker for monitoring disease.

Our results support earlier studies showing that a type-I IFN signature is a key feature of JDM [4, 14–16, 37]. While past research found higher IFN scores in active disease, our data show that certain IRGs are clearly linked to specific disease areas. Strong negative correlations with CMAS and MMT8 highlight the close link between IFN pathway activation and muscle inflammation. Similarly, strong links with skin disease activity, including classic rashes and vasculopathic features, show that IFN-driven inflammation affects more than just muscles.

In contrast, conventional laboratory markers of inflammation, including ESR, CRP and CK, showed inconsistent associations with disease activity, in keeping with their recognized limited sensitivity in JDM. These correlations should therefore be interpreted with caution and appeared less informative than IFN-related signatures. Associations between IRG expression and haematological parameters, such as anaemia and lymphopenia, may also have been confounded by immunosuppressive treatment rather than reflecting disease activity. In addition, variable corticosteroid exposure across patients and timepoints may have influenced IRG expression and represents a potential limitation of the longitudinal analyses.

A key finding is that IRG expression differs between organs. The type-II IRG *CXCL9* was linked to ILD, suggesting that IFN-γ-driven macrophage activation contributes to lung involvement. In contrast, *IL-18* was strongly associated with calcinosis, skin ulcers and gastrointestinal problems, pointing to its role in ongoing tissue damage and inflammation. These results align with new data showing *IL-18* is involved in severe inflammation in other rheumatic and autoinflammatory diseases [38, 39]. This suggests that non-traditional IFN-related mediators can add useful information to the classic type-I IFN signature. Overall, while type-I IFN activation is a general marker of inflammation, type-II-related mediators like *CXCL9* and *IL-18* may help identify specific, clinically important subtypes of JDM.

Looking at IRG expression by MSA status shows that autoantibodies in JDM reflect the underlying immune system, and not just act as clinical markers. Anti-MDA5 and anti-NXP2 antibodies identified a group with high IFN and increased IRG expression, matching clinical features like vasculopathy, ulcers, lung disease and widespread inflammation. In contrast, patients with anti-TIF1γ and anti-Mi2 had lower IFN signatures, supporting the idea that these groups are biologically different. These results support a precision-medicine approach, where MSA status helps classify immune subtypes and IFN signatures give more detail for risk and disease monitoring.

Our analyses over time show that IRG expression in JDM changes with disease activity. Patients who improved had lower levels of key IFN-stimulated genes, supporting the idea that IFN activation reflects current immune activity rather than a lasting disease marker. The drop in IRG expression after baricitinib treatment supports this, showing that IFN pathways can be directly affected and that IFN signatures can be used as biomarkers for tracking targeted therapies. While these findings support biological responsiveness, formal evaluation of sensitivity to change and minimal clinically important differences will require prospectively designed studies.

Modelling patient data over time showed clear groups based on changes in IFN levels. These different patterns could help predict disease progression, identify children at risk of ongoing inflammation and guide long-term monitoring. While more studies are needed to confirm this, these results suggest that tracking IFN signatures could help guide clinical decisions about treatment.

Adding IRG expression to disease assessment could help overcome some of the limitations of current monitoring. Clinical scores and functional tests are important, but they can vary between observers and may miss hidden immune activity. IFN-based tests, especially those targeting key genes such as *SIGLEC1* and *IFI27*, could provide objective, reliable measures to complement current tools and precision medicine in JDM.

This study has several strengths. The main one is combining detailed IRG expression data with comprehensive clinical information across multiple organ systems in a large cohort of children with JDM. The rich dataset, including detailed skin findings, gut involvement, lab markers, autoantibody subgroups and changes over time, enabled us to connect IFN biology to the complex clinical picture of JDM, a rare disease in rare disease research. Collection of samples overtime gave insight into how IFN activation changes, showing patterns of remission and treatment response that support IFN signatures as dynamic biomarkers. Studying both type-I and type-II IFN-related mediators also helped identify different molecular subtypes that could be important for personalized monitoring and future targeted treatments.

There are some limitations to note. Although this is a large cohort for a rare disease, recruitment from a single tertiary centre may limit how widely applicable the results are and could bias the sample towards more severe or complex cases. The dataset is detailed, but still too small to study rare disease features, which is common in rare disease research. The real-world data are both a strength and a weakness: they reflect everyday clinical practice and patient differences, but samples were not always collected at set times and follow-up varied in length and frequency. As a result, the analyses over time were based on a subset of patients with repeated measurements, with different numbers of samples per person, which may have weakened some findings and given more weight to patients with more frequent sampling. In addition, latent class trajectory analyses were anchored to the first available IRG sampling timepoint rather than to disease onset, which may limit interpretation of these groups as true disease evolution phenotypes. Finally, the baricitinib analyses should be interpreted cautiously given the observational design, the small treated subgroup, heterogeneous sampling intervals and potential treatment indication bias and therefore require validation in prospectively designed studies.

These analyses were exploratory and included many correlation tests across clinical and molecular markers, which could increase the chance of type-I error. However, this was done on purpose to find biologically plausible links and generate ideas for future studies, not to create final predictive models. Also, using gene expression from blood is practical, but it may not fully represent disease processes in muscle, skin or lung tissue. Testing these findings in other patient groups and combining them with protein tests, functional studies and other molecular approaches will be important next steps to improve biomarker use and understand the mechanisms involved.

Since IRG expression is being considered as a possible biomarker in JDM, it is important to validate its use. For reliability, IFN signature tests are technically consistent and have been used in several centres with standard procedures, and central lab analysis is available when needed [40, 41]. Although this study did not formally test repeatability within individuals, earlier research has shown that IFN signatures are stable over short periods, supporting their use in research and clinical care [41, 42]. For validity, there is no gold-standard molecular test for inflammation in JDM, but our results give early evidence of convergent validity, as IFN signatures matched up logically with clinical measures, organ-specific features and autoantibody subgroups. While this dataset was not designed to test predictive validity or treatment response systematically, the changes in IFN signatures observed with clinical improvement and targeted therapy are promising. Future studies with planned sampling and clear objectives will be needed to confirm predictive value and treatment response, and to decide how best to use IFN-based biomarkers in disease monitoring.

In summary, our data show that IRG expression, mainly from type-I IFN-inducible genes but also including type-II-related mediators, provides a clear molecular framework linking disease activity, organ involvement and immune subtypes in JDM. The way IFN signatures change with disease and treatment supports their use as practical biomarkers. These results lay the groundwork for using IFN profiling in personalized disease monitoring and treatment planning in JDM.

## Supplementary Material

keag384_Supplementary_Data

## Data Availability

De-identified datasets and analytical protocols are available upon request from the corresponding author.
