# Supplementary material for: IFN-related gene expression defines disease activity, organ involvement and treatment response in JDM
Source: Rheumatology (Oxford). 2026 Jul 25;65(8):keag384. doi: 10.1093/rheumatology/keag384 (PMC13428460; doi:10.1093/rheumatology/keag384)
Supplement: keag384_Supplementary_Data [file keag384_supplementary_data.docx]

**Supplementary material**

**Supplementary tables**

Supplementary Table S1. Associations between Interferon-related gene expression and specific cutaneous manifestations in our JDM cohort

| **Cutaneous feature** | **IFI27 (p)** | **IFI44L (p)** | **IFIT1 (p)** | **IL-18 (p)** | **RSAD2 (p)** | **SIGLEC1 (p)** | **Others (p)** |
| --- | --- | --- | --- | --- | --- | --- | --- |
| Gottron’s sign | 0.003 | 0.002 | 0.006 | 0.011 | 0.004 | 0.001 | - |
| Heliotrope rash | 0.032 | 0.003 | 0.002 | 0.001 | 0.008 | 0.005 | - |
| Periungual erythema | 0.021 | 0.004 | 0.008 | 0.042 | 0.014 | 0.004 | - |
| Non-sun-exposed erythema | 0.002 | 0.010 | 0.038 | 0.001 | 0.010 | 0.020 | - |
| Malar/facial erythema | 0.001 | 0.001 | 0.000 | N/S | 0.003 | 0.000 | - |
| Extensive erythema | N/S | N/S | N/S | 0.016 | N/S | N/S | - |
| “V” sign | 0.018 | N/S | N/S | N/S | N/S | N/S | - |
| Shawl sign | 0.000 | 0.001 | 0.005 | N/S | 0.001 | 0.001 | - |
| Periungual capillary loop changes | N/S | 0.048 | 0.024 | 0.032 | N/S | N/S | IFNG: 0.037 |
| Cutaneous ulceration | N/S | N/S | 0.039 | 0.046 | 0.023 | N/S | - |
| Calcinosis | N/S | N/S | N/S | 0.030 | N/S | N/S | - |

*p: p-value (statistical significance defined as <0.05)*

Supplementary Table S2. Comparison of Interferon-related gene expression values between patient classes

| **IRG** | **Kruskal-Wallis p-value** | **Classes compared** | **Dunn's pairwise test adjusted p-value** |
| --- | --- | --- | --- |
| CXCL10 | 0.1270987993 | N/A | N/A |
| CXCL9 | 0.3281311610 | N/A | N/A |
| IFI27 | 0.0000095995 | 1 - 2 | 0.0000948184 |
| IFI27 |  | 1 - 3 | 0.0000165158 |
| IFI27 |  | 2 - 3 | 0.0247152663 |
| IFI44L | 0.0000219707 | 1 - 2 | 0.0004454241 |
| IFI44L |  | 1 - 3 | 0.0000175041 |
| IFI44L |  | 2 - 3 | 0.0120300052 |
| IFIT1 | 0.0001815912 | 1 - 2 | 0.0004539308 |
| IFIT1 |  | 1 - 3 | 0.0003175947 |
| IFIT1 |  | 2 - 3 | 0.0681587110 |
| IFNB1 | 0.6257694509 | N/A | N/A |
| IFNG | 0.2955232306 | N/A | N/A |
| IL18 | 0.2026820772 | N/A | N/A |
| RSAD2 | 0.0000364467 | 1 - 2 | 0.0002728743 |
| RSAD2 |  | 1 - 3 | 0.0000479345 |
| RSAD2 |  | 2 - 3 | 0.0288192912 |
| SIGLEC1 | 0.0003482383 | 1 - 2 | 0.0011007218 |
| SIGLEC1 |  | 1 - 3 | 0.0003925369 |
| SIGLEC1 |  | 2 - 3 | 0.0512228142 |
| IFN score | 0.0000603144 | 1 - 2 | 0.0008450355 |
| IFN score |  | 1 - 3 | 0.0000441347 |
| IFN score |  | 2 - 3 | 0.0152794147 |

*N/A: Not Applicable*

Supplementary Table S3. Comparison of interferon-related gene expression between on-treatment and off-treatment samples

| **IRG** | **Man-Whitney p-value**  **(Bonferoni adjusted p-value)** | **Treatment Group**  **Median** | **No-Treatment Group**  **Median** |
| --- | --- | --- | --- |
| CXCL10 | 0.523794636 (1) | 0.98 | 1.02 |
| CXCL9 | 0.103107454 (1) | 0.40 | 0.47 |
| IFI27 | 0.172340302 (1) | 6.78 | 2.72 |
| IFI44L | 0.698588542 (1) | 2.35 | 1.21 |
| IFIT1 | 0.773844448 (1) | 2.39 | 1.44 |
| IFNB1 | 0.01578579 (0.173643691) | 0.23 | 0.33 |
| IFNG | 0.414622812 (1) | 0.35 | 0.25 |
| IL18 | 0.360794898 (1) | 0.70 | 0.77 |
| RSAD2 | 0.998102829 (1) | 2.00 | 1.00 |
| SIGLEC1 | 0.679404718 (1) | 0.34 | 0.33 |
| IFN score | 0.78598818 (1) | 0.81 | 0.78 |

Supplementary Table S4. Longitudinal changes in interferon-related gene expression after baricitinib initiation

| **IRG** | **Man-Whitney p-value**  **(adjusted p-value)** | **Before Baricitinib**  **Median** | **After Baricitinib**  **Median** |
| --- | --- | --- | --- |
| CXCL10 | 0.278320313 (1) | 2.46 | 0.88 |
| CXCL9 | 0.683480902 (1) | 0.38 | 0.32 |
| IFI27 | 0.000976563 (0.010742188) | 106.42 | 13.85 |
| IFI44L | 0.001953125 (0.021484375) | 13.77 | 2.51 |
| IFIT1 | 0.002929688 (0.032226563) | 15.08 | 3.60 |
| IFNB1 | 0.004882813 (0.053710938) | 0.43 | 0.24 |
| IFNG | 0.13047493 (1) | 0.21 | 0.42 |
| IL18 | 0.221271816 (1) | 0.83 | 0.58 |
| RSAD2 | 0.001953125 (0.021484375) | 16.95 | 2.00 |
| SIGLEC1 | 0.000976563 (0.010742188) | 8.99 | 0.40 |
| IFN score | 0.000976563 (0.010742188) | 3.10 | 0.87 |
